# Gastrointestinal dysfunction is associated with mortality in severe burn patients: a 10-year retrospective observational study from South China

**DOI:** 10.1186/s40779-022-00403-1

**Published:** 2022-09-05

**Authors:** Qiu-Lan He, Shao-Wei Gao, Ying Qin, Run-Cheng Huang, Cai-Yun Chen, Fei Zhou, Hong-Cheng Lin, Wen-Qi Huang

**Affiliations:** 1 Department of Anesthesiology, the First Affiliated Hospital of Sun Yat-sen University, Guangzhou, 510080 China; 2 Department of Anesthesiology, Zhongshan People’s Hospital, Zhongshan, 528400 Guangdong China; 3 Department of Anesthesiology, Dongguan People’s Hospital, Dongguan, 523059 Guangdong China; 4 Department of Burn Surgery, the First Affiliated Hospital of Sun Yat-sen University, Guangzhou, 510080 China; 5 Department of Colorectal Surgery, the Sixth Affiliated Hospital of Sun Yat-sen University, Guangzhou, 510655 China

**Keywords:** Severe burn, Gastrointestinal dysfunction, Mortality, Sepsis, Gastrointestinal haemorrhage, Continuous analgesia

## Abstract

**Background:**

Data on severe and extensive burns in China are limited, as is data on the prevalence of a range of related gastrointestinal (GI) disorders [such as stress ulcers, delayed defecation, opioid-related bowel immotility, and abdominal compartment syndrome (ACS)]. We present a multicentre analysis of coincident GI dysfunction and its effect on burn-related mortality.

**Methods:**

This retrospective analysis was conducted on patients with severe [≥ 20% total burn surface area (TBSA)] and extensive (> 50% TBSA or > 25% full-thickness TBSA) burns admitted to three university teaching institutions in China between January 1, 2011 and December 31, 2020. Both 30- and 90-day mortality were assessed by collating demographic data, burn causes, admission TBSA, % full-thickness TBSA, Baux score, Abbreviated Burn Severity Index (ABSI) score, and Sequential Organ Failure Assessment (SOFA) score, shock at admission and the presence of an inhalation injury. GI dysfunction included abdominal distension, nausea/vomiting, diarrhoea/constipation, GI ulcer/haemorrhage, paralytic ileus, feeding intolerance and ACS. Surgeries, length of intensive care unit (ICU) stay, pain control [in morphine milligram equivalents (MME)] and overall length of hospital stay (LOHS) were recorded.

**Results:**

We analyzed 328 patients [75.6% male, mean age: (41.6 ± 13.6) years] with a median TBSA of 62.0% (41.0–80.0%); 256 (78.0%) patients presented with extensive burns. The 90-day mortality was 23.2% (76/328), with 64 (84.2%) of these deaths occurring within 30 d and 25 (32.9%) occurring within 7 d. GI dysfunction was experienced by 45.4% of patients and had a significant effect on 90-day mortality [odds ratio (*OR*) = 14.070, 95% confidence interval (CI) 5.886–38.290, *P* < 0.001]. Multivariate analysis showed that GI dysfunction was associated with admission SOFA score and % full-thickness TBSA. Overall, 88.2% (67/76) of deceased patients had GI dysfunction [hazard ratio (*HR*) for death of GI dysfunction = 5.951], with a survival advantage for functional disorders (diarrhoea, constipation, or nausea/vomiting) over GI ulcer/haemorrhage (*P* < 0.001).

**Conclusion:**

Patients with severe burns have an unfavourable prognosis, as nearly one-fifth died within 90 d. Half of our patients had comorbidities related to GI dysfunction, among which GI ulcers and haemorrhages were independently correlated with 90-day mortality. More attention should be given to severe burn patients with GI dysfunction.

**Supplementary Information:**

The online version contains supplementary material available at 10.1186/s40779-022-00403-1.

## Background

Burn injuries are common traumatic injuries, with an estimated 6 million patients worldwide seeking medical attention for burns each year [1, 2]. The cellular and sub-cellular pathophysiology of severe burn injury is complex, with systemic effects on organ systems and changes resulting from inflammation, hyper-metabolism, catabolic muscle wasting and insulin resistance [3, 4]. In critical burns, there is a panoply of general gastrointestinal (GI) dysfunction syndromes, which include delayed defecation [5, 6], opioid-related bowel dysfunction [7, 8], acute colonic pseudo-obstruction [9], abdominal compartment syndrome (ACS) [10, 11], and acute mesenteric ischaemia [12]. The development of these complaints can contribute to systemic inflammatory response syndrome (SIRS) and multiple organ dysfunction syndrome (MODS). The pathogenesis of GI dysfunction involves increased gastric secretion, reduced intestinal motility, impaired nutrient absorption, enhanced mucosal permeability, bacterial translocation effects, alterations in intra-abdominal pressure, and in some cases, a severe disturbance of GI barrier function [5, 13, 14].

Early fluid resuscitation, continuous renal replacement therapy, selective administration of low-dose dopamine to dilate mesenteric vasculature and early enteral feeding have all contributed to improvements in the clinical outcome of severe burns [15–19]. Over the past decade, these advances have improved the overall survival rate of severe burn patients; however, the incidence of cases with severe GI dysfunction remains stubbornly high [20]. Trexler et al. [6] conducted a retrospective analysis of patients admitted to a specialized burn intensive care unit (ICU); these patients had > 20% total burn surface area (TBSA), required mechanical ventilation, and some (36.1%) cases had delayed defecation as defined by the absence of defecation > 6 d after admission. These patients were more likely to have episodes of constipation following their first defecation as well as feeding intolerance that required more frequent parenteral nutrition. Similarly, in a prospective observational study recently reported by Strang et al. [21], the prevalence of intra-abdominal hypertension was as high as 53% in patients with > 15% TBSA burns admitted to two burn units in the Netherlands.

The significance of these and other complications, such as pressure ulcer development, impacts both the length of ICU stay in severe burn patients and mortality rates [22, 23]. Gut-related sepsis and MODS can still ensue even when the early associated GI dysfunction is relatively mild and reversible. These effects may be related to a dysbiosis with overgrowth of harmful bacteria due to the breakdown of the normal intestinal barrier and/or altered immune responsiveness [24, 25]. Moreover, the role of selective decontamination of the bowel or the use of pre- or pro-biotics and their effects on mucosal immunology and clinical outcomes in critical burn patients are yet to be adequately determined [26, 27]. We present the results of a retrospective multicentre study evaluating the prevalence and nature of GI dysfunction in a population of severe burn patients and report the association between objective GI dysfunction and mortality.

## Methods

### Patients selection and records extraction

This retrospective study was conducted with data from the databases of the First Affiliated Hospital of Sun Yat-sen University (FAH), the Zhongshan People’s Hospital (ZPH) and the Dongguan People’s Hospital (DPH) collected between January 1, 2011 and December 31, 2020. The study was mainly organized as a case–control design, which compared and assessed the clinicopathological features of different stratifications such as survival/deceased or with/without GI dysfunction, as well as investigated prognostic factors for patients admitted with severe burns. The protocol of the study and analysis was approved by the local hospital ethics committees (FAH-2021-014, ZPH-K2021-049, and DRYA-2021-054-A1). Patients included in this study were adults > 18 years of age with > 20% TBSA; patients who arrived > 72 h after their burn injury or who died within 48 h of admission were excluded from analysis. The primary outcome measure was 90-day mortality, with the secondary outcome measuring the incidence of GI dysfunction. Demographic data (i.e., age and sex) were collated along with the cause of the burn. Admission data included the TBSA (in accordance with the Lund-Brower chart), the % full-thickness TBSA, the Baux score mortality predictor, the Abbreviated Burn Severity Index (ABSI), the Sequential Organ Failure Assessment (SOFA) score, the presence of sepsis according to Sepsis-3.0 (evidence of infection combined with a SOFA score of 2 points or more [28–31]), the presence of associated trauma and shock at admission and the presence of an inhalation injury. Mental symptoms within 1 month after admission (i.e., early mental symptoms) were also extracted and recorded from the electronic daily medical records, including emotional symptoms (sadness, anxiety, irritability, etc.), cognitive symptoms (confusion, memory impairment, etc.), perceptual symptoms (hallucinations) and behavioral symptoms (provocation, self-mutilation, insomnia, etc.).

### Determination of GI dysfunction of severe burn patients

GI dysfunction was mainly determined by manually reviewing each patient’s detailed course records, the evaluation time window encompassed the entire treatment process since the patient’s hospitalization after the burn. Evidence of GI dysfunction was considered on clinical grounds with symptoms including abdominal distension, abdominal pain, nausea, vomiting, failure to have a bowel movement for > 6 d after admission or > 3 bowel movements per day. Supportive testing for the diagnosis of GI dysfunction included, where appropriate, an abdominal computed tomography (CT) scan, positive endoscopy, occult blood positivity (vomitus or stools) and corroborative laboratory testing. Complications such as paralytic ileus, pneumatosis intestinalis and GI perforation were all recorded based on electronic medical records. To establish a diagnosis, GI dysfunction was evaluated by a gastroenterologist. In detail, GI haemorrhage was defined when an asymptomatic patient had continuous positive results on the occult blood test throughout 1 week or direct evidence of GI haemorrhage was viewed with an endoscopy. Paralytic ileus, pneumatosis intestinalis and GI perforation were determined according to plain abdominal radiography, dynamic measurement of abdominal circumference and intra-abdominal pressure. To facilitate the analysis, GI dysfunction was then stratified as GI haemorrhage or disturbed GI motility consisting of nausea/vomiting, abdominal distension, constipation and diarrhoea. Patients with both GI haemorrhage and disrupted GI motility were categorized in the GI haemorrhage group because it has a worse prognosis. Additionally, a sensitivity analysis was used to check if the results changed when the patients with both GI haemorrhage and disrupted GI motility were excluded.

### Clinical interventions of severe burn patients

Interventions included fluid resuscitation; a range of surgical procedures (escharotomy, fasciotomy, skin grafting); the use of antacids, probiotics and/or vasopressors; enteral and parenteral feeding; and a variety of analgesic medications. The length of ICU stay and the length of hospital stay (LOHS) were recorded along with the incidence of sepsis and MODS. All patients enrolled received fluid resuscitation in accordance with the Army Military Medical University formula for intravenous fluids, a resuscitative regime widely used throughout China for managing severe burn patients within 48 h of hospital admission [32]. In brief, the total volumes of colloid and crystalloid in the 24 h are calculated based on a 1.5 ml/(kg‧%) TBSA with a crystalloid: colloid ratio 2:1 plus 2000 ml of 5% glucose solution as physiological requirement. In general, tracheostomies were used in patients with deep circumferential neck burns, where there were symptoms of airway obstruction (change in voice, stridor or laryngeal dyspnoea) and suspicion (or evidence) of inhalation injury. Protective ventilation was initiated when appropriate to maintain an inspiratory plateau pressure < 30 cmH_2_O. In general, the three burn centres aimed to commence enteral nutrition (EN) within 12–24 h after admission where possible, given the benefit of enteral feeding to GI barrier function recovery. Patients with more extensive burns (> 50% TBSA or > 25% full-thickness TBSA) commenced enteral feeding within 24–72 h after admission. In the early phase, short peptides were favoured with < 30% lipids per total caloric intake plus glutamine and probiotic (live combined *Bifidobacterium* and *Lactobacillus* tablets) supplementation. Patients routinely received proton pump inhibitors (PPIs; most commonly omeprazole) by injection. Surgical treatments included, where necessary, early escharotomy or fasciotomy with early autograft or allograft coverage (within 7 d of admission) of excised burn wounds.

### Statistical analysis

Data were analysed using Statistical Product Service Solutions (version 23; SPSS Inc., Chicago, IL, USA) and R software (version 4.0.5). Continuous data are presented as the mean ± standard deviation (SD) or *M* (*Q*_1_, *Q*_3_) where appropriate. Student’s *t* test was used to compare normally distributed continuous data, and nonparametric analyses included the Mann–Whitney *U* and Kruskal–Wallis tests. Categorical variables were expressed as *n* (%), and the Shapiro–Wilk test was used to assess the normality of the data. Categorical data were compared using the chi-square test or Fisher’s exact test where appropriate. Stepwise logistic regression analysis was performed to determine variables with significant risk for GI dysfunction and for 30- and 90-day burn mortality. For further analysis where time-to-event data were used as the outcome, log-rank test was used to compare Kaplan–Meier curves and Cox proportional hazards regression was used to multivariately assess predictors of outcome. Variables entered into the risk model for GI dysfunction included the % TBSA of the burns, the % full-thickness TBSA, the admission Baux score, the admission SOFA score, the admission ABSI score, the presence of any inhalation injury, the MODS, the presence of sepsis and the use of continuous analgesia; less than 5% of these variables were missing values and they were directly removed before modelling. Variables included in the assessment of mortality were patient age, admission complicated by shock, length of ICU stay, total lymphocyte count after the first surgery, MME required per day during the ICU stay, occurrence of wound sepsis and presence of GI dysfunction. For all methods above, *P* < 0.05 was considered statistically significant.

## Results

### Patient characteristics

**Fig. 1:**
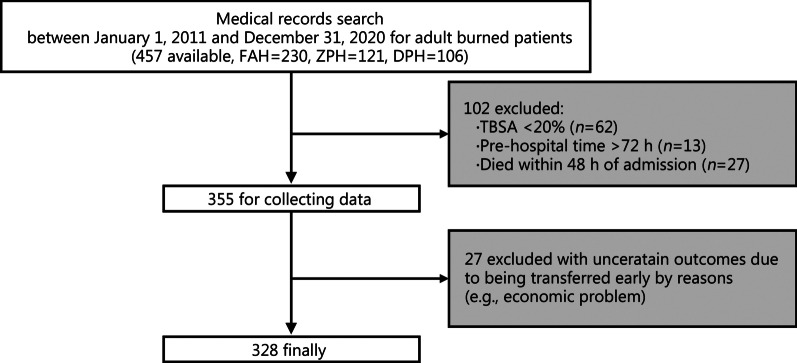
Flowchart of the study. FAH the First Affiliated Hospital of Sun Yat-sen University, ZPH Zhongshan People’s Hospital, DPH Dongguan People’s Hospital, TBSA total body surface area

During the study period, a total of 457 adult burn patients were admitted to the three participating hospitals; 355 severe burn patients fulfilled the inclusion and exclusion criteria for analysis. Of these, 27 patients were excluded due to uncertain outcomes because of early transfers for reasons such as economic problems, and thus data collation was incomplete. These patients were excluded from the analysis, for a final sample size of 328 patients and a nearly complete dataset during follow-up (recruitment rate: 82.8%). Figure 1 shows the flow chart for the study.

There was a preponderance of male patients (248/328, 75.6%) with a median overall age of (41.6 ± 13.6) years and a median TBSA of 62.0% (41.0–80.0%). Two hundred and fifty-six patients (78.0%) presented with extensive burns (% TBSA > 50%, or % full-thickness TBSA > 20% or combined with severe inhalation injury). Of these cases, 110 required ICU admission. There were 45 patients (13.7%) with pre-existing chronic comorbidities and 5 patients (1.5%) with an underlying systemic GI disorder (3 with chronic constipation and 2 with chronic gastritis). During the study period, 130 patients (39.6%) received non-intraoperative mechanical ventilation, all of whom had complications of inhalation injury and head and face burns. Additionally, 178 patients (54.3%) received continuous analgesia, 145 patients (44.2%) were treated with continuous intravenous analgesia (CIA), and 33 (10.1%) received prescribed patient-controlled analgesia (PCA).

### GI dysfunction is associated with 90-day mortality

Ninety days after admission for burn injury, 76 patients (23.2%) had died, with 64 (84.2%) of these deaths occurring within 30 d and 25 (32.9%) occurring within 7 d. Table 1 shows the characteristics of 76 non-survivors and 252 survivors. Over the study period, 12–75 severe burn admissions were registered per annum with a peak in 2018 and a peak mortality (38.6%, 22/57) in 2019. Table 2 shows the factors associated with survival after 90 d. There was a significant effect of inhalation injury (*r* = 0.285, *P* < 0.001) or shock at admission (*r* = 0.227, *P* < 0.001), % TBSA (*r* = 0.477, *P* < 0.001), % full-thickness TBSA (*r* = 0.473, *P* < 0.001), Baux score (*r* = 0.487, *P* < 0.001), ABSI score (*r* = 0.493, *P* < 0.001), length of ICU stay (*r* = 0.499, *P* < 0.001), MODS (*r* = 0.662, *P* < 0.001), sepsis (*r* = 0.483, *P* < 0.001), associated GI dysfunction (*r* = 0.471, *P* < 0.001), wound infection (*r* =—0.127, *P* = 0.022), early mental symptoms (*r* = 0.276, *P* = 0.025) and continuous analgesia (*r* = 0.185, *P* < 0.001).

**Table 1:** Patient characteristics of severe burn included in this study

Item	Non-survivor (*n* = 76)	Survivor (*n* = 252)	*P*-value
Age (years, mean ± SD)	44.3 ± 12.4	40.8 ± 13.8	0.045
Weight (kg, mean ± SD)	66.3 ± 10.2	66.2 ± 12.4	0.929
Sex [*n*(%)]			0.888
Male	57 (75.0)	191 (75.8)	
Female	19 (25.0)	61 (24.2)	
Injury source [*n*(%)]			0.215
Fire	63 (82.9)	180 (71.4)	
Chemicals	8 (10.5)	45 (17.9)	
Hot liquid	2 (2.6)	16 (6.3)	
Electrical	3 (3.9)	11 (4.4)	
Principally burned region [*n*(%)]			< 0.001
Head/face/neck	73 (96.1)	209 (82.9)	
Hand	69 (90.8)	199 (79.0)	
Perineum	35 (46.1)	37 (14.7)	
rVAS [*M* (*Q*_1_, *Q*_3_)]	4 (3, 5)	5 (4, 6)	0.064
WBC [× 10^9^/L, *M* (*Q*_1_, *Q*_3_)]	18.7 (13.1, 24.0)	13.0 (9.8, 18.4)	< 0.001
CRP [mg/ml, *M* (*Q*_1_, *Q*_3_)]	41.0 (8.0, 116.5)	65.1 (29.7, 117.7)	0.287
Platelet [× 10^9^/L, *M* (*Q*_1_, *Q*_3_)]	237 (141, 353)	200 (124, 295)	0.022
ALB [g/L, *M* (*Q*_1_, *Q*_3_)]	30.6 (23.0, 38.6)	28.3 (25.0, 33.6)	0.370
LOHS [d, *M* (*Q*_1_, *Q*_3_)]	13 (7, 26)	49 (33, 77)	< 0.001

*SD* standard deviation, *rVAS* baseline resting Visual Analogue Scale (VAS) of pain at admission, *WBC* white blood cell, *CRP* C-reactive protein, *ALB* albumin, *LOHS* length of hospital stay

**Table 2:** Factors associated with 90-day mortality in severe burn patients

Item	Non-survivor (*n* = 76)	Survivor (*n* = 252)	*r*	*P*-value
Inhalation injury [*n*(%)]			0.285	< 0.001
Yes	66 (86.8)	136 (54.0)		
No	10 (13.2)	116 (46.0)		
Shock at admission [*n*(%)]			0.227	< 0.001
Yes	46 (60.5)	87 (34.5)		
No	30 (39.5)	165 (65.5)		
Incidence separated on % TBSA [*n*(%)]			0.470	< 0.001
20–29	2 (2.6)	19 (7.5)		
30–49	1 (1.3)	76 (30.2)		
50–69	12 (15.8)	81 (32.1)		
70–89	27 (35.5)	60 (23.8)		
≥ 90	34 (44.7)	16 (6.3)		
% TBSA [*M* (*Q*_1_, *Q*_3_)]	85 (75, 95)	55 (35, 70)	0.477	< 0.001
% full-thickness TBSA [*M* (*Q*_1_, *Q*_3_)]	55 (36, 78)	15 (3, 34)	0.473	< 0.001
Baux score [*M* (*Q*_1_, *Q*_3_)]	143 (129, 157)	106 (84, 127)	0.487	< 0.001
SOFA score [*M* (*Q*_1_, *Q*_3_)]	2 (1, 5)	2 (1, 3)	0.106	0.056
ABSI score [*M* (*Q*_1_, *Q*_3_)]	14 (13, 15)	11 (9, 14)	0.493	< 0.001
Length of ICU stay [d, *M* (*Q*_1_, *Q*_3_)]	5 (1, 12)	0 (0, 0)	0.499	< 0.001
MODS [*n*(%)]			0.662	< 0.001
Yes	61 (80.3)	26 (10.3)		
No	15 (19.7)	226 (89.7)		
Sepsis^*^ [*n*(%)]			0.483	< 0.001
Yes	56 (73.7)	51 (20.2)		
No	20 (26.3)	201 (79.8)		
GI dysfunction [*n*(%)]			0.471	< 0.001
Yes	67 (88.2)	82 (32.5)		
No	9 (11.8)	170 (67.5)		
Wound infection [*n*(%)]			− 0.127	0.022
Yes	14 (18.4)	79 (31.3)		
No	62 (81.6)	168 (66.7)		
Early mental symptoms [*n*(%)]			0.276	0.025
Emotional symptoms	11 (14.5)	23 (9.1)		
Perceptual symptoms	4 (5.3)	11 (4.4)		
Cognitive disorder	10 (13.2)	16 (6.3)		
Behavioral disorder	15 (19.7)	21 (8.3)		
None	58 (76.3)	219 (86.9)		
Continuous analgesia with opioids [*n*(%)]			0.185	< 0.001
Yes	54 (71.1)	124 (49.2)		
No	22 (28.9)	128 (50.8)		

*TBSA* total body surface area, *SOFA* Sequential Organ Failure Assessment, *ABSI* Abbreviated Burn Severity Index, *ICU* intensive care unit, *MODS* multiple organ dysfunction syndrome, *GI* gastrointestinal

^*^Sepsis was diagnosed according to the definition of Sepsis 3.0 (2016)

Table 3 shows the causes of death in the patient dataset, with early deaths mainly due to respiratory or cardiac causes and late deaths largely a result of septic shock. A multiple logistic regression analysis of the entire dataset correctly identified 93.1% of the 90-day mortalities (Nagelkerke’s *R*^*2*^ = 0.622) and detected 4 correlative factors (Table 4). There was a negative influence of the % full-thickness TBSA [odds ratio (*OR*) = 1.039, 95% confidence interval (CI) 1.024–1.056, *P* < 0.001], sepsis (*OR* = 9.241, 95% CI 4.211–21.600, *P* < 0.001) and GI dysfunction (*OR* = 14.070, 95% CI 5.886–38.290, *P* < 0.001). In this analysis, the presence of GI dysfunction had the greatest effect on 90-day mortality. Receiving continuous analgesia was associated with a lower 90-day predicted mortality (*OR* = 0.477, 95% CI 0.238–0.904, *P* = 0.029).

**Table 3:** Causes of severe burn patient death [*n*(%)]

Category	Total (*n* = 76)	< 30-day mortality (*n* = 63)	30- to 90-day mortality (*n* = 13)
Respiratory			
Pneumonia/ARDS	11 (14.5)	9 (14.3)	2 (15.4)
Airway obstruction	6 (7.9)	6 (9.5)	0
Respiratory failure	11 (14.5)	10 (15.9)	1 (7.7)
Cardiovascular			
Septic shock	19 (25.0)	13 (20.6)	6 (46.1)
Cardiac shock	6 (7.9)	6 (9.5)	0
Hypovolemic shock	8 (10.5)	8 (12.7)	0
Gastrointestinal			
Ischaemic bowel	4 (5.3)	2 (3.2)	2 (15.4)
Haemorrhage	4 (5.3)	4 (6.3)	0
Metabolic			
Hypernatremia	2 (2.6)	2 (3.2)	0
Severe acidemia	1 (1.3	1 (1.6)	0
Unknown	4 (5.3)	2 (3.2)	2 (15.4)

*ARDS* acute respiratory distress syndrome

**Table 4:** Factors associated with 90-day mortality in patients with severe burns (*n* = 328)

Variable	*OR*	95% CI	*P*-value
% full-thickness TBSA	1.039	1.024–1.056	< 0.001
Shock at admission	2.173	1.000–4.806	0.051
Sepsis^*^	9.241	4.211–21.600	< 0.001
GI dysfunction	14.070	5.886–38.290	< 0.001
Continuous analgesia	0.477	0.238–0.904	0.029

*TBSA* total body surface area, *GI* gastrointestinal

^*^Sepsis was diagnosed according to the definition of Sepsis 3.0 (2016)

### GI haemorrhage rather than motility problem is associated with increased mortality

In the patient dataset, 149 (45.4%) developed GI dysfunction with a diverse range of problems, including GI haemorrhage or ulcer (Additional file 1: Fig. S1) in 45 (30.2%) patients, nausea and vomiting in 33 (22.1%) patients, delayed defecation in 64 (43.0%) patients, abdominal distention in 27 (18.1%) patients, and diarrhoea in 8 (5.4%) patients. Overall, 67 of the 76 (88.2%) patients who died had some form of GI dysfunction. The recorded incidence of GI dysfunction in severe burn cases between 2011 and 2020 fluctuated between 5/20 (25.0%) in 2015 and 11/18 (61.1%) in 2011. The correlative percentage of deaths in patients with GI dysfunction also varied between 1/8 (12.5%) in 2013 and 18/28 (64.3%) in 2019. The univariate analysis of the variables potentially associated with GI dysfunction is summarized in Table 5*,* showing an influence of inhalation injury; shock at admission; % TBSA and % full-thickness TBSA; Baux, SOFA and ABSI scores at admission and MME. Multivariate analysis showed that GI dysfunction was independently affected by the % full-thickness TBSA (*OR* = 1.020, 95% CI 1.011–1.030, *P* < 0.001), the SOFA score at admission (*OR* = 1.197, 95% CI 1.066–1.350, *P* = 0.003) and accompanying early mental symptoms (*OR* = 2.758, 95% CI 1.373–5.796, *P* = 0.005) (Additional file 2: Table S1). Table 6 shows a subgroup analysis of specific GI complications that were significantly associated with unfavourable outcomes (90-day mortality, MODS, sepsis and length of ICU stay). These included GI haemorrhage or ulcers and nausea or vomiting but not constipation or abdominal distension.

**Table 5:** Factors associated with the occurrence of GI dysfunction in patients with severe burns

Variable	GI dysfunction (*n* = 149)	Non-GI dysfunction (*n* = 179)	*P*-value
Age (years, mean ± SD)	41.8 ± 14.2	41.4 ± 13.0	0.819
Weight (kg, mean ± SD)	66.1 ± 11.1	66.3 ± 12.7	0.920
Sex [*n*(%)]			0.865
Male	112 (75.2)	136 (76.0)	
Female	37 (24.8)	43 (24.0)	
Injury source [*n*(%)]			0.228
Fire	112 (75.2)	131 (73.2)	
Chemicals	26 (17.4)	27 (15.1)	
Hot liquid	4 (2.7)	14 (7.8)	
Electricity	7 (4.7)	7 (3.9)	
Inhalation injury [*n*(%)]			< 0.001
Yes	112 (75.2)	90 (50.3)	
No	37 (24.8)	89 (49.7)	
Trauma at admission [*n*(%)]			0.374
Yes	12 (8.1)	10 (5.6)	
No	137 (91.9)	169 (94.4)	
Shock at admission [*n*(%)]			0.017
Yes	71 (47.7)	62 (34.6)	
No	78 (52.3)	117 (65.4)	
Incidence separated on % TBSA [*n*(%)]			< 0.001
20–29	9 (6.0)	12 (6.7)	
30–49	19 (12.8)	58 (32.4)	
50–69	36 (24.2)	57 (31.8)	
70–89	49 (32.9)	38 (21.2)	
≥ 90	36 (24.2)	14 (7.8)	
% TBSA [*M* (*Q*_1_, *Q*_3_)]	75 (52, 89)	52 (35, 70)	< 0.001
% full-thickness TBSA [*M* (*Q*_1_, *Q*_3_)]	35 (14, 59)	14 (2, 34)	< 0.001
Baux score [*M* (*Q*_1_, *Q*_3_)]	128 (102, 146)	105 (83,127)	< 0.001
SOFA score [*M* (*Q*_1_, *Q*_3_)]	3 (1, 5)	2 (1, 3)	< 0.001
ABSI score [*M* (*Q*_1_, *Q*_3_)]	11 (9, 13)	10 (8, 12)	0.023
WBC [× 10^9^/L, *M* (*Q*_1_, *Q*_3_)]	16.7 (10.3, 22.3)	13.1 (10.0, 18.1)	0.009
CRP [mg/ml, *M* (*Q*_1_, *Q*_3_)]	57.0 (18.8, 112.0)	56.8 (23.5, 117.8)	0.679
Platelet [× 10^9^/L, *M* (*Q*_1_, *Q*_3_)]	205 (117, 301)	203 (131, 306)	0.752
ALB [g/L, *M* (*Q*_1_, *Q*_3_)]	28.0 (23.8, 34.6)	29.1 (25.6, 36.0)	0.048
Sepsis^*^ [*n*(%)]			< 0.001
Yes	68 (45.6)	39 (21.8)	
No	81 (54.4)	140 (78.2)	
Wound infection [*n*(%)]			0.227
Yes	38 (25.5)	55 (30.7)	
No	111 (74.5)	119 (66.5)	
High MME requirement [*n*(%)]			0.283
Yes	31 (20.8)	29 (16.2)	
No	118 (79.2)	150 (83.8)	
MME [mg, *M* (*Q*_1_, *Q*_3_)]	196.0 (70.0, 506.0)	138.0 (47.5, 348.8)	0.010
Continuous analgesia with opioids [*n*(%)]			0.078
CIA	74 (49.7)	71 (39.7)	
PCA	17 (11.4)	16 (8.9)	
None	58 (38.9)	92 (51.4)	

*GI* gastrointestinal, *SD* standard deviation, *TBSA* total body surface area, *SOFA* Sequential Organ Failure Assessment, *ABSI* Abbreviated Burn Severity Index, *WBC* white blood cell, *CRP* C-reactive protein, *ALB* albumin, *MME* morphine milligram equivalent, *CIA* continuous intravenous analgesia, *PCA* patient-controlled analgesia

^*^Sepsis was diagnosed according to the definition of Sepsis 3.0 (2016)

**Table 6:** Subgroup analysis of the association between GI dysfunction and clinical outcomes in patients with severe burns

Clinical outcome	Victims^*^	Controls^**^	*P*-value
GI haemorrhage or ulcer (*n*_victims_ = 45, *n*_controls_ = 283)			
90-day mortality [*n*(%)]	34 (75.6)	42 (14.8)	< 0.001
MODS [*n*(%)]	35 (77.8)	52 (18.4)	< 0.001
Sepsis [*n*(%)]	28 (62.2)	79 (27.9)	< 0.001
Length of ICU stay [d, *M* (*Q*_1_, *Q*_3_)]	4 (0, 10)	0 (0, 3)	< 0.001
Nausea/vomiting (*n*_victims_ = 33, *n*_controls_ = 295)			
90-day mortality [*n*(%)]	15 (45.5)	61 (20.7)	0.004
MODS [*n*(%)]	15 (45.5)	72 (24.4)	0.013
Sepsis [*n*(%)]	19 (57.6)	88 (29.8)	0.003
Length of ICU stay [d, *M* (*Q*_1_, *Q*_3_)]	2 (0, 10)	0 (0, 3)	0.003
Constipation (*n*_victims_ = 64, *n*_controls_ = 264)			
90-day mortality [*n*(%)]	20 (31.3)	56 (21.2)	0.099
MODS [*n*(%)]	19 (29.7)	68 (25.8)	0.530
Sepsis [*n*(%)]	21 (32.8)	86 (32.6)	0.971
Length of ICU stay [d, *M* (*Q*_1_, *Q*_3_)]	0 (0, 5)	0 (0, 4)	0.590
Abdominal distension (*n*_victims_ = 27, *n*_controls_ = 301)			
90-day mortality [*n*(%)]	18 (66.7)	58 (19.3)	< 0.001
MODS [*n*(%)]	11 (40.7)	76 (25.2)	0.109
Sepsis [*n*(%)]	13 (48.1)	94 (31.2)	0.087
Length of ICU stay [d, *M* (*Q*_1_, *Q*_3_)]	0 (0, 10)	0 (0, 4)	0.115

*GI* gastrointestinal, *MODS* multiple organ dysfunction syndrome, *ICU* intensive care unit

^*^Victims refer to patients suffering the corresponding subsymptoms of GI dysfunction

^**^Controls refer to the remaining patients other than the patients with the specified GI dysfunction subsymptoms; they may suffer other subsymptoms of GI dysfunction

**Fig. 2:**
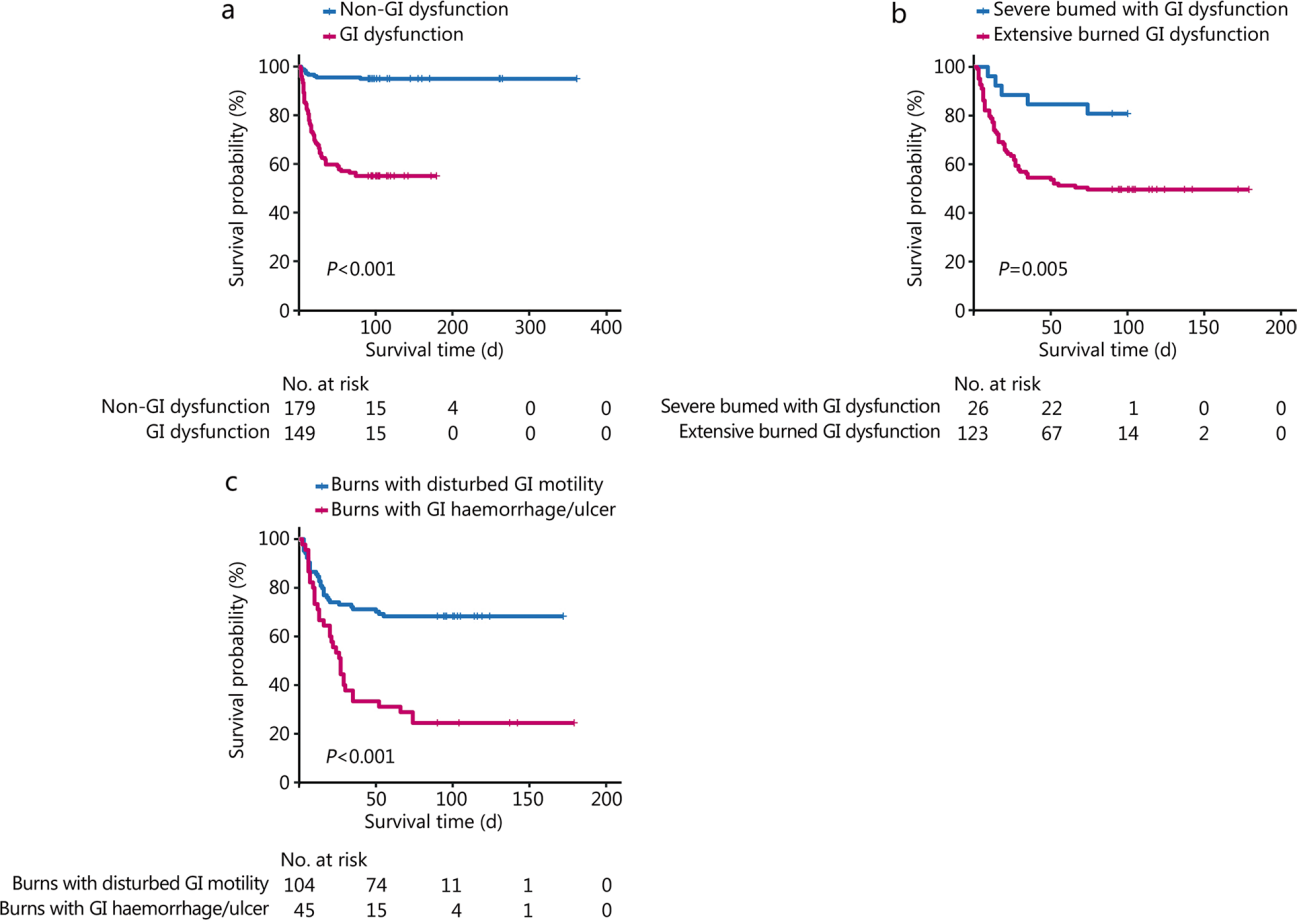
Kaplan–Meier survival curves of burn patients with GI dysfunction in multi-level comparisons. **a** Kaplan–Meier survival curves of patients admitted with severe burns: the effect of GI dysfunction. **b** Kaplan–Meier survival curves: severe versus extensive burns (> 50% TBSA or > 20% full-thickness TBSA). **c** Kaplan–Meier survival curves: GI haemorrhage/ulcer patients vs. those with a GI motility disorder (constipation/diarrhoea, nausea/vomiting, or abdominal distension). GI gastrointestinal, TBSA total body surface area

The 30-day mortality was significantly greater in patients with GI dysfunction than in those without GI dysfunction (87.5% vs*.* 12.5%, *χ*^2^ = 58.8, *P* < 0.001). This effect extended to the 90-day mortality comparisons between the GI dysfunction and non-GI dysfunction groups (88.2% vs*.* 11.8%, *χ*^2^ = 72.9, *P* < 0.001), as 170/179 patients (95.0%) without GI dysfunction survived. Figure 2a shows the Kaplan–Meier overall survival curves with a significant difference between patients with and without associated GI dysfunction (*P* < 0.001). After eliminating the likely effects of variables selected for their predictive impact on mortality, Cox regression analysis showed an influence of the % full-thickness TBSA, the presence of sepsis, GI dysfunction and the need for continuous analgesia on survival. The adjusted hazard ratio (*HR*) for death in patients with GI dysfunction was 5.951 (95% CI 2.900–12.213, *P* < 0.001) and 3.182 (95% CI 1.811–5.589, *P* < 0.001) for patients with associated sepsis (Table 7).

**Table 7:** Cox regression analysis of the effect of GI dysfunction on survival of severe burns (*n* = 328)

	*HR*	95% CI	*P*-value
% full-thickness TBSA	1.024	1.014–1.033	< 0.001
Shock at admission	1.556	0.945–2.562	0.083
Sepsis^*^	3.182	1.811–5.589	< 0.001
GI dysfunction	5.951	2.900–12.213	< 0.001
Continuous analgesia—CIA	1.463	0.871–2.458	0.150
Continuous analgesia—PCA	0.251	0.085–0.741	0.012

*TBSA* total body surface area, *GI* gastrointestinal, *CIA* continuous intravenous analgesia, *PCA* patient-controlled analgesia

^*^Sepsis was diagnosed according to the definition of Sepsis 3.0 (2016)

In the observational study, 24 patients (15 males) were diagnosed with ACS based upon signs, symptoms and a measured intra-abdominal pressure exceeding 20 mmHg. In this ACS group, only 9 patients (37.5%) survived; most of them (8/9) received continuous analgesia. To further assess survival, the entire dataset was divided into 2 subgroups based on burn severity. This included those with severe burns (as defined) and those with extensive burns (cases with > 50% TBSA or > 20% full-thickness TBSA). Figure 2b shows the significantly shorter median survival in patients with extensive burns than that in patients with severe burns (55 d vs*.* 74 d, *P* = 0.005). Figure 2c separates patients with GI dysfunction into those with haemorrhage and those with motility problems (constipation/diarrhoea, nausea/vomiting or abdominal distension). The pattern of results remained the same when patients with both haemorrhage and mobility disorders were removed (Additional file 1: Fig. S2). Kaplan–Meier analysis showed a survival advantage for patients with any kind of motility disorder over patients with GI ulcer/haemorrhage (*P* < 0.001). In patients with GI ulcer/haemorrhage, 50% mortality occurred within 27 d post-admission.

## Discussion

There was a significant impact of TBSA and severity scores on mortality, with nearly half the patients presenting with an associated GI dysfunction in this study. Notably, GI dysfunction was implicated in 88.2% of the deaths, specifically if it manifested as GI haemorrhage or nausea and vomiting but not if there were predominant functional symptoms such as constipation and/or diarrhoea. In general, GI dysfunction was more common in patients with an inhalation injury, shock at admission, a greater TBSA and a high morphine requirement (Table 5).

Collated worldwide studies assessing epidemiological changes in burn admissions over time tend to demonstrate a downwards trend in total burn injuries, which is predominantly found in countries with improvements in overall socioeconomic status over recent decades. These demographic changes are usually (but not always) accompanied by a reduction in burn-related mortality and LOHS [2]. Burn data concerning Chinese patients are limited, with some studies reporting no significant changes over time in burn severity [33]. These findings have not generally been accompanied by an equivalent reduction in overall burn severity when the number of elderly patients presenting with severe burns as a proportion of the total number of severe burns cases has increased. Similar findings to ours have been reported in China by Cheng et al. [34] in a multicenter observational study showing the particular importance of inhalation injury on mortality in extensive burn cases. Demographic comparisons of burn care need to be made with caution since there will be differences in the number of elderly patients along with variation in the cause and severity of burns.

The factors that affect severe burn admissions differ across countries and reflect the robust nature of some legislative changes, community-based preventative programs and workplace safety. The expansion of urbanization of rural areas in China and the development of its social economy have extended the types of burn-related lifestyle factors normally seen in urban residents to rural environments [35]. Generally, in China, the outcomes of severe burns depend on the % full-thickness TBSA, patient age and comorbidity; in our data, the principal effects of these factors on burn-related mortality reflected the severity of the burn at admission. This is consistent with the results of previous studies, such as that of Tian et al. [36], who also noted correlations of age, the presence of an inhalation injury, TBSA and % full-thickness TBSA on mortality in severe burn patients. Similar findings have been reported in Europe [1], Australia [37] and Malaysia [38].

GI dysfunction with severe burns is common, but depending upon its definition, reports of the incidence of a panoply of conditions that include stress ulceration, nausea and vomiting, generalized and specific motility disturbances, ACS and nutrient malabsorption vary. The most severe GI dysfunction results in disruption of the mucosal barrier, which is directly implicated in SIRS and enhanced endotoxin absorption and bacterial translocation that led to MODS and death [13, 39, 40]. In our study, we found that if TBSA increased, so did the incidence of GI dysfunction and mortality (Tables 2, 5). Similar to our study, Ng et al. [8] found that the majority of patients with severe burns who subsequently died had some form of GI dysfunction and that GI dysfunction was more likely in those cases admitted with an inhalation injury, shock at admission or with a larger TBSA. However, whether GI dysfunction contributes to the mechanism by which higher TBSA leads to increased mortality is unknown. Some evidence indicates that increased TBSA induces earlier onset of hypovolemic shock, which is refractory and has systemic effects on organs of all body systems, including reduction in venous return to the heart (preload), and resulting in further gut ischaemia and oedema [41]. A vicious cycle develops, with the destruction of the intestinal barrier and the decline in absorption capacity further worsening the negative nitrogen balance, and thus significantly increasing the risk of MODS and mortality. Future studies should focus on the role of GI dysfunction in the lethal mechanism of high TBSA.

In addition to TBSA, GI dysfunction was also associated with sepsis (Spearman’s *r* = 0.237, *P* < 0.001) (Table 6, Additional file 2: Table S2), but sepsis was not an independent risk factor for GI dysfunction in the multiple logistic regression analysis. The iSOFA study indicated that the Gastrointestinal Dysfunction Score (GIDS) combined with the SOFA score could better predict 28-day and 90-day mortality of critically ill patients than using a single SOFA score [42]. We found similar results, where the retrospectively scored GI dysfunction of our patients before or within 1 month after injury were strongly correlated with all-cause 90-day mortality, both in univariate and multivariate analyses (*P* < 0.001, Additional file 2: Table S3). However, common severity scoring systems [e.g., Acute Physiology and Chronic Health Evaluation (APACHE) and SOFA] rarely assess GI dysfunction. As increasing evidence highlights the importance of GI dysfunction, its prognostic value should be considered when updating these scoring systems in the future.

As noted in our study, the major mortality risk in severe burn patients may be more related to GI haemorrhage from a stress-induced (Curling’s) ulcer rather than any functional disturbance, such as constipation or delayed defecation, usually consequent upon feeding intolerance. Although clinically significant stress ulceration is less common, it is associated with an increase in mortality [43]. The underlying pathophysiology of GI haemorrhages is unclear, although hypoxia and hypoperfusion are likely to be the most important factors. Once intestinal haemorrhage occurs, the loss of epithelial cells in the corresponding parts of the intestine suggests the alteration of intestinal permeability, destruction of the gut vascular barrier and an increased risk of bacterial translocation [44]. In addition, repeated and massive GI haemorrhages further increase the risk of blood volume loss and even aspiration, which will cause secondary injury to severe burns and increase the risk of death. Recent GI dysfunction papers [42, 45] have shown that GI bleeding is higher-level evidence of GI injury in critically ill patients, which strongly suggests GI failure.

In contrast, the pathogenesis and significance of delayed defecation in these patients are less clear, although it may signify a global GI dysmotility that presents as a symptomatic constellation that also includes feeding intolerance, appetite suppression, opioid-induced bowel dysfunction and electrolyte imbalance. In the Fukuda et al. [46] study, delayed defecation was also associated with the time to weaning from a ventilator or coincident sepsis, both of which were also closely correlated with the length of ICU stay. All of these factors, however, were viewed as markers of disease severity as there is currently no consensus regarding the definition of GI dysfunction or failure in severely burned cases [47]. Critically ill patients suffering constipation are more likely to fail at oral feeding or fail to wean early from a mechanical ventilator, suggesting that there is a clinical effect of delayed defecation on hospital outcome [48]. Notably, although there is no association reported in severe burns between delayed defecation and mortality [5, 6, 23] and most of the symptoms of GI dysfunction were also mild in this study, once two or three intractable symptoms developed in the short term, the risk of death was significantly increased from 37.7% (46/122) to 77.8% (21/27). Finally, nearly two-thirds of patients with ACS died. Although the incidence of ACS in severe burn patients is low, it correlates with TBSA and is a harbinger of MODS and mortality [10, 11]. This would suggest the value of more routine pressure measurement [21].

Successfully managing GI dysfunction can be a challenge in burn patients when compounded by impaired mucosal absorption, excessive fluid losses, prolonged immobility, sepsis and the effects of repeated surgery. If the gut is malfunctioning, parenteral nutrition is the obvious choice for nutritional supplementation [49]. However, although parenteral nutrition for severe burn patients is easier to deliver, it has more complicated metabolic consequences, which are considered harmful in critically ill patients with intense inflammation [50]. EN, especially early EN (starting within 12–24 h after the burn), may play a role in preventing GI injury after severe burns. Early EN stimulates intestinal contraction through direct contact between the nutrients and intestinal mucosal cells and is of great importance in weakening hypermetabolism, reducing circulating stress hormones, and protecting the integrity and function of the intestinal mucosa. A large number of studies have provided evidence that early EN reduces the risk of GI bleeding, sepsis and organ injury, shortening the LOHS [51]. Raff et al. [52] reported that the risk of GI haemorrhage in severe burn patients treated with early EN was reduced by 40%. However, the optimal EN intervention time is still controversial, and precise evaluation tools that can be popularized in clinics are lacking. As this was a retrospective study, it is difficult to accurately determine the specific timing of EN and to trace the dynamic changes in nutritional data. After reviewing data from the three enrolled hospitals, we found that many patients had received treatment in local hospitals for hours to days before admission. During the prehospital period, the main treatment was anti-shock and life support. Therefore, some patients may fail to start early EN (within 24 h after burn) and suffer a higher risk of mortality. When patients developed EN intolerance, downregulation or even suspension of EN and parenteral nutrition supplementation was the first consideration. For intractable symptoms, prokinetics or antidiarrhoeal agents were added, and the nutrition specialist took part in adjusting the nutrition plan.

The intestinal microbiota of severe burn patients was seriously altered, especially non-surviving patients with MODS, who had marked and continuous microbiota alterations. Shimizu et al. [53] observed the abundance of intestinal microbiota in patients with major burns and found that among non-survivors, the number of *Bifidobacteria* decreased significantly, while the number of *Pseudomonas* and *Candida* increased markedly. The balance of the intestinal microbiota was destroyed, increasing the concentration of total organic acids in the faeces and significantly reducing the concentration of beneficial short-chain fatty acids (SCFAs), such as acetic acid, propionic acid and butyric acid. Previous study has shown that a decrease in SCFA levels is related to susceptibility to inflammation in severe burn patients [54]. In addition, the long-term and high-dose use of antibiotics will also aggravate the imbalance of intestinal flora and may increase the risk of *Clostridium difficile* infection [55]. Although there was no positive detection of *Clostridium difficile*, at least in the blood samples in our data (Additional file 2: Table S4), we indeed found that a large portion of patients had an imbalance in the faecal *Coccus*-to-*Bacillus* (C/B) ratio, which may be attributed to *Clostridium difficile*. Therefore, maintaining the balance of intestinal microbiota promotes the function of the immune system. In the three hospitals included in this study, probiotics have become the routine treatment for patients with severe burns. However, in patients with intestinal dyskinesia, such as patients with EN intolerance, oral administration of probiotics may not achieve intestinal production. Because intestinal barrier disruption may originate from multiple complex factors [25, 56], an overall treatment method must be adopted, including the combination of early EN, active fluid resuscitation, antacids, prokinetic drugs and probiotics.

Pain management is a central component of the treatment of burn patients [57, 58]. In China, in the past decade, a growing number of burn and pain specialists have gradually realized the importance of analgesia in controlling excessive stress in severe burn patients and have prescribed zero/low opioid background intravenous PCA for them [59, 60]. In this study, we found that opioid PCA or intermittent opioid analgesia (rather than continuous intravenous injection of opioids) at a constant rate is safer and associated with more survival benefits in severe burn patients. Possible mechanisms are control of the stress exacerbated by moderate to severe pain, reduction of excessive stress hormone levels, and improvements to organ function [26, 61].

Our study has several limitations. The study was retrospective in design and limited to only 3 institutions. Strict comparisons with other studies are difficult since burn mortality depends upon the number of elderly patients, who have generally higher ABSI and Baux scores, more comorbidities (hypertension, diabetes, chronic obstructive pulmonary disease), and specific cause of the burns (more explosions, flames, scalding). The improvements in the burn mortality of elderly cases over time have been fairly modest, suggesting that this is more an effect of changes in burn severity in this group rather than a result of treatment modifications [2].

## Conclusions

In conclusion, patients with severe burns often have an unfavourable prognosis, with one-third admitted to the ICU and one-fifth of burn-related deaths occurring early in treatment. Half of the severe burn patients and most of the patients who subsequently died had GI dysfunction where mortality was correlated with GI ulcers/haemorrhages rather than functional GI presentations. The admission criteria, along with the presence of GI dysfunction, therefore predict mortality risk in severe burn patients admitted to an ICU.

## Supplementary Information

**Additional file 1: Fig. S1**. Endoscopy images of gastric haemorrhage/ulcer in patients with extensive burns. **Fig. S2**. Kaplan–Meier survival curves: exclusive GI haemorrhage/ulcer (without a motility disorder) vs. GI motility disorder (constipation/diarrhoea, nausea/vomiting, or abdominal distension).

**Additional file 2: Table S1**. Factors associated with GI dysfunction in patients with severe burns (*n* = 328). **Table S2**. Correlations between GI dysfunction and discrete clinical variables (*n* = 149). **Table S3**. Factors associated with 90-day mortality in patients with severe burns (*n* = 328). **Table S4**. Summary of blood pathogens detected in severe burn patients with bacteremia [a total of 102 positive blood cultures were reported in 91 patients, *n*(%)].

## Data Availability

The datasets generated during and/or analysed during the current study are not publicly available due to they contain the personal health information and important privacy of each patient, but are available from the corresponding author (Wen-Qi Huang) on reasonable request.

## Abbreviations

ABSI: Abbreviated Burn Severity Index
ACS: Abdominal compartment syndrome
APACHE: Acute Physiology and Chronic Health Evaluation
C/B: *Coccus*-to-*Bacillus*
CT: Computed tomography
CI: Confidence interval
CIA: Continuous intravenous analgesia
DPH: Dongguan People’s Hospital
EN: Enteral nutrition
FAH: The First Affiliated Hospital of Sun Yat-sen University
GI: Gastrointestinal
GIDS: Gastrointestinal Dysfunction Score
HR: Hazard ratio
ICU: Intensive care unit
LOHS: Length of hospital stay
MME: Morphine milligram equivalents
MODS: Multiple organ dysfunction syndrome
OR: Odds ratio
PCA: Patient-controlled analgesia
PPIs: Proton pump inhibitors
SCFAs: Short-chain fatty acids
TBSA: Total burn surface area
SD: Standard deviation
SOFA: Sequential Organ Failure Assessment
SIRS: Systemic inflammatory response syndrome
ZPH: Zhongshan People’s Hospital
