# Supplementary material for: Gastrointestinal dysfunction is associated with mortality in severe burn patients: a 10-year retrospective observational study from South China
Source: Mil Med Res. 2022 Sep 5;9:49. doi: 10.1186/s40779-022-00403-1 (PMC9442990; doi:10.1186/s40779-022-00403-1)
Supplement: Supplementary file 1 — Additional file 1: Fig. S1. Endoscopy images of gastric haemorrhage/ulcer in patients with extensive burns. Fig. S2. Kaplan–Meier survival curves: exclusive GI haemorrhage/ulcer (without a motility disorder) vs. GI motility disorder (constipation/diarrhoea, nausea/vomiting, or abdominal distension). [file 40779_2022_403_MOESM1_ESM.pdf]

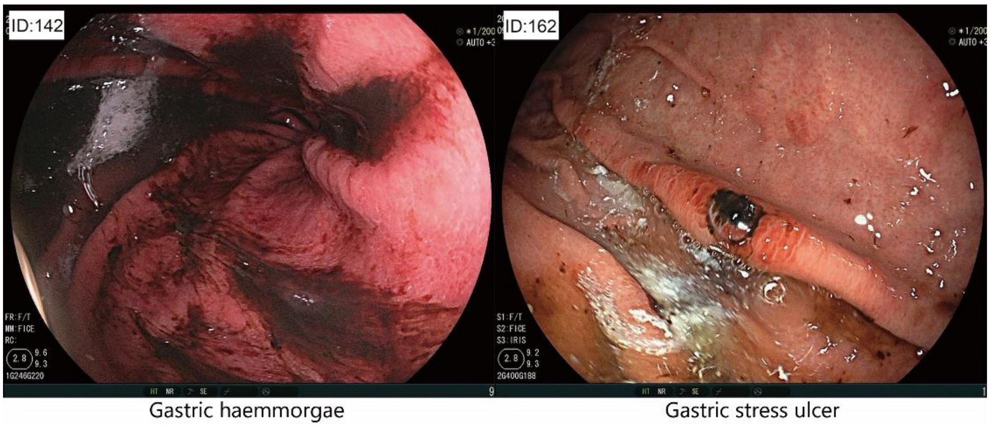

Fig. S1 Endoscopy images of gastric haemorrhage/ulcer in patients with extensive burns

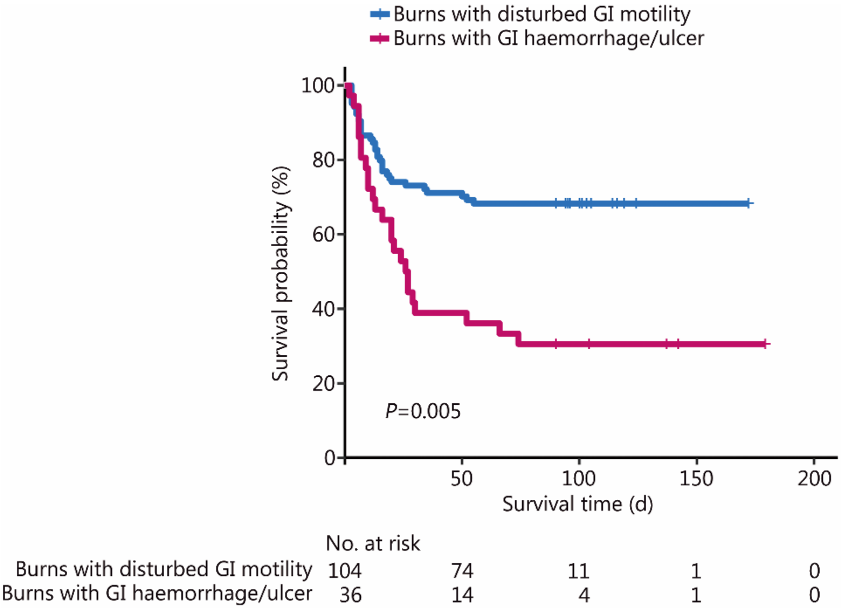

Fig. S2 Kaplan–Meier survival curves: exclusive GI haemorrhage/ulcer (without a motility disorder) vs. GI motility disorder (constipation/diarrhoea, nausea/vomiting, or abdominal distension). GI gastrointestinal
