# Supplementary material for: Gastrointestinal dysfunction is associated with mortality in severe burn patients: a 10-year retrospective observational study from South China
Source: Mil Med Res. 2022 Sep 5;9:49. doi: 10.1186/s40779-022-00403-1 (PMC9442990; doi:10.1186/s40779-022-00403-1)
Supplement: Supplementary file 2 — Additional file 2: Table S1. Factors associated with GI dysfunction in patients with severe burns (n = 328). Table S2. Correlations between GI dysfunction and discrete clinical variables (n = 149). Table S3. Factors associated with 90-day mortality in patients with severe burns (n = 328). Table S4. Summary of blood pathogens detected in severe burn patients with bacteremia [a total of 102 positive blood cultures were reported in 91 patients, n(%)]. [file 40779_2022_403_MOESM2_ESM.pdf]

Table S1 Factors associated with GI dysfunction in patients with severe burns (n = 328)

Variable	*OR*	95%CI	*P*-value
% full-thickness TBSA	1.020	1.011 – 1.030	< 0.001
SOFA at admission	1.197	1.066 – 1.350	0.003
Early mental symptoms	2.758	1.373 – 5.796	0.005

GI gastrointestinal, *TBSA* total body surface area, *SOFA* Sequential Organ Failure Assessment

Table S2 Correlations between GI dysfunction and discrete clinical variables (*n* = 149)

Discrete clinical variables	Spearman's *r*	95%CI	*P*-value
Shock at admission	0.138	0.027 – 0.246	0.013
Sepsis	0.237	0.128 – 0.340	< 0.001
Continuous analgesia	0.103	-0.009 – 0.213	0.064
Bacteremia	0.174	0.064 – 0.280	0.002

GI gastrointestinal

Table S3 Factors associated with 90-day mortality in patients with severe burns (n = 328)

Variable	*OR*	95%CI	*P*-value
% full-thickness TBSA	1.043	1.024 – 1.065	< 0.001
Shock at admission	3.743	1.474 – 10.12	0.007
Sepsis	16.71	6.202 – 52.54	< 0.001
GIDS*	6.337	3.915 – 11.29	< 0.001
Continuous analgesia	0.461	0.204 – 0.965	0.049

*GIDS is used in place of gastrointestinal dysfunction. *TBSA* total body surface area, *GIDS* Gastrointestinal Dysfunction Score (0 – 4 points)

Table S4 Summary of blood pathogens detected in severe burn patients with bacteremia [a total of 102 positive blood cultures were reported in 91 patients, *n*(%)]

Type of pathogen	Bacteremia (*n* = 102)	Mortality of bacteremia
Bacteria		
Acinetobacter baumannii	56 (54.9)	24 (42.9)
Pseudomonas aeruginosa	28 (27.5)	14 (50.0)
Klebsiella pneumoniae	15 (14.7)	5 (33.3)
Staphylococcus aureus	13 (12.7)	2 (15.4)
Enterococcus faecalis	8 (7.8)	3 (37.5)
Stenotrophomonas maltophilia	11 (10.8)	2 (18.2)
Escherichia coli	10 (9.8)	5 (50.0)
Enterobacter cloacae	4 (3.9)	2 (50.0)
Fungus		
Candida parapsilosis	8 (7.8)	3 (37.5)
Candida albicans	2 (2.0)	0
Aspergillus	2 (2.0)	2 (100.0)
Others	15 (14.7)	4 (26.6)
